# Changes in the Repertoire of tRNA-Derived Fragments in Different Blood Cell Populations

**DOI:** 10.3390/life14101294

**Published:** 2024-10-12

**Authors:** Alexander A. Artamonov, Kirill A. Kondratov, Egor A. Bystritsky, Yuri V. Nikitin, Anastasiya A. Velmiskina, Sergey V. Mosenko, Irina A. Polkovnikova, Anna Yu. Asinovskaya, Svetlana V. Apalko, Natalya N. Sushentseva, Andrey M. Ivanov, Sergey G. Scherbak

**Affiliations:** 1 City Hospital No. 40, St. Petersburg 197706, Russia; artamonov.alandr@yandex.ru (A.A.A.);; 2 Kirov Military Medical Academy, St. Petersburg 194044, Russia; dr.iuriinikitin@gmail.com (Y.V.N.);; 3 Saint-Petersburg State University, St. Petersburg 199034, Russia; 4 ITMO University, St. Petersburg 197101, Russia; ertalt333@gmail.com

**Keywords:** tRNA fragment, blood cells, severe COVID-19, sRNA NGS, RBC, lymphocytes, monocytes

## Abstract

tRNA-derived fragments function as markers in addition to playing the key role of signalling molecules in a number of disorders. It is known that the repertoire of these molecules differs greatly in different cell types and varies depending on the physiological condition. The aim of our research was to compare the pattern of tRF expression in the main blood cell types and to determine how the composition of these molecules changes during COVID-19-induced cytokine storms. Erythrocytes, monocytes, lymphocytes, neutrophils, basophils and eosinophils from control donors and patients with severe COVID-19 were obtained by fluorescence sorting. We extracted RNA from FACS-sorted cells and performed NGS of short RNAs. The composition of tRNA-derived fragments was analysed by applying a semi-custom bioinformatic pipeline. In this study, we assessed the length and type distribution of tRFs and reported the 150 most prevalent tRF sequences across all cell types. Additionally, we demonstrated a significant (*p* < 0.05, fold change >16) change in the pattern of tRFs in erythrocytes (21 downregulated, 12 upregulated), monocytes (53 downregulated, 38 upregulated) and lymphocytes (49 upregulated) in patients with severe COVID-19. Thus, different blood cell types exhibit a significant variety of tRFs and react to the cytokine storm by dramatically changing their differential expression patterns. We suppose that the observed phenomenon occurs due to the regulation of nucleotide modifications and alterations in activity of various Rnases.

## 1. Introduction

tRNA-derived fragments (tRFs) are a relatively recently discovered class of small RNA molecules that are shown to be involved in the pathogenesis of various diseases, particularly cancer and viral infections. Processed from transfer RNAs, tRFs regulate gene expression through various mechanisms like miRNA-like silencing of target mRNAs [1,2,3,4].

There are several types of classified tRFs based on the cleavage site within the tRNA molecule: tRF-1—Cleaved from the 3′ trailer sequence of pre-tRNAs; tRF-2—Cleaved from the D-loop of mature tRNAs; tRF-3—Cleaved from the 3′ end of mature tRNAs; tRF-5—Cleaved from the 5′ end of mature tRNAs, i-tRF—Internal tRFs derived from the internal region of mature tRNAs [5].

Different tissues and organs possess a different set of tRNA-derived fragments [2]. Nevertheless, it has not been studied how the pattern of these molecules differs in different blood cells. Supposably, the repertoire of these molecules changes in correspondence with changes in the immunological state of patients. In particular, it is not clear how these molecules will change in the blood cells of patients during a COVID-19-induced cytokine storm.

Additionally, tRFs have been implicated in multiple aspects of cancer biology:

Regulation of Cell Viability: They can influence tumour cell proliferation, differentiation, and apoptosis [6].

Gene Regulation: tRFs participate in post-transcriptional regulation, acting similarly to microRNAs by binding to target mRNAs, thereby affecting gene expression [5].

Biomarkers for Diagnosis and Prognosis: Altered levels of specific tRFs have been associated with various cancers, suggesting their utility as diagnostic or prognostic biomarkers [2].

It has been previously reported by Wu et al. that tRFs were the most significantly affected small non-coding RNAs in nasopharyngeal swabs of COVID-19 patients; they had also observed that SARS-CoV-2-infected airway epithelial cells exhibit the same tendency [4]. In an additional attempt to shed light on tRNA-derived fragments and their involvement in host–virus interactions, it seems relevant to study tRFs in different pathophysiological circumstances. In the case of this study, our aim was to reveal the differential expression of tRFs in fluorescence-activated sorted cells of healthy control donors and SARS-CoV-2-infected patients, as well as to observe and characterise tRFs during viral infections.

## 2. Materials and Methods

### 2.1. Patients and Data Collection

Six healthy donors and five RT-PCR-confirmed SARS-CoV-2-positive patients made up the two groups of research participants. Three patients were hospitalised to the critical care unit, and two patients were admitted to the infectious disease unit. Severe patients satisfied the following criteria for admission to the intensive care unit (ICU): body temperature ≥39 °C, respiratory rate ≥30/min, and oxygen saturation (SpO2) ≤93%. The St. Petersburg City Hospital No. 40 Expert Ethics Council authorised the research (protocol No. 171 of 18 May 2020).

### 2.2. Cell Sorting

Cell sorting was performed according to a standardised technique [7].

Erythrocyte sorting was carried out as follows:

A mixture of 100 µL PBS, 10 µL whole blood, 2 µL anti-cd235 (IM2212U), and 2 µL cd41 (A07781) was used. Fluorescent sorting was performed up to 5 million events using a MoFlo Astrios EQ flow cytometer sorter (fitted with 405, 488, and 645) after the sample was dissolved in 1 millilitre of phosphate-buffered saline (PBS) and incubated for 20 min in the dark.

Leukocyte sorting was performed using the following two distinct techniques:

Each tube containing 100 µL of heparinised peripheral blood was filled with 1 mL of VersaLyse lysing solution (Beckman Coulter, Brea, CA, USA), shaken, and left to incubate for 10 min. To differentiate between lymphocytes and monocytes, 10 µL of the relevant monoclonal antibodies were added: CD45-APC-AlexaFluor^®^ 750+ (A79392 Beckman Coulter, Brea, CA, USA), CD16-PC7+ (6607118 Beckman Coulter, Brea, CA, USA), and CD14-PC5.5+ (A70204 Beckman Coulter, Brea, CA, USA).

The samples were vortex-mixed and left at room temperature in the dark for 15 to 20 min. One millilitre of phosphate-buffer saline (PBS) was then added, and each tube was centrifuged for five minutes at 1500 rpm. The supernatant was then discarded, and one millilitre of buffer solution was added. Utilising a MoFlo Astrios EQ flow cytometer sorter (fitted with 405, 488, and 645 lasers), the resuspended pellet was examined. The granulocyte subpopulations, eosinophils, basophils, and neutrophils, were isolated using the duraclone IM granulocyte antibody panel (B88651) from Beckman Coulter, USA.

The cells were sorted into sterile 1.5 mL tubes using nozzles with a diameter of 70 μm, with 50,000–100,000 events for the leukocyte cell population.

**Figure 1:**
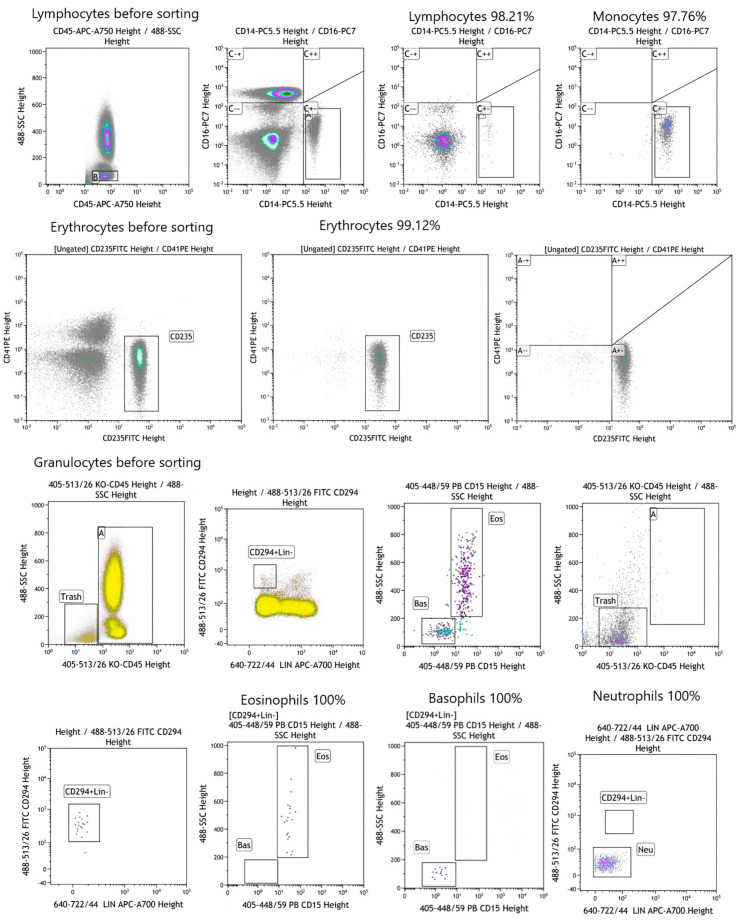
Erythrocytes, monocytes, lymphocytes, neutrophils, basophils, and eosinophils from peripheral blood were sorted using fluorescence-activated cell sorting. Verification of the sorted cell populations’ purity.

Flow cytometry data showed that the purity of the cell samples was >95% for all cell types (Figure 1).

### 2.3. RNA Separation, Library Preparation and Next-Generation Sequencing

RNA was isolated or extracted using ExtractRNA reagent (Evrogen, Moscow, Russia) according to the provided protocol. After extraction, RNA was dissolved in 10 µL RNAse-free water. The quality of the obtained RNA was checked on a TapeStation (Agilent, Santa Clara, CA, USA). The concentration of RNA, dsDNA, and ssDNA was evaluated on Quantus dual-channel fluorometer (Promega, Madison, WI, USA) using the following kits: QuantiFluor^®^ RNA System kit (E3310 Promega, Madison, WI, USA), QuantiFluor^®^ dsDNA System (E2671, Promega, Madison, WI, USA), QuantiFluor^®^ ssDNA System (E3190, Promega, Madison, WI, USA). Only samples with RIN diapason between 5 and 8 were taken for sequencing. Short RNA libraries were prepared using the Small RNA Library Prep Kit (1000006383 MGI, Shenzhen, China). After preparation, libraries were purified with electrophoresis in a 6% polyacrylamide gel. A 100–135 bp band was excised from the gel, which corresponds to an RNA length of 17–50 bp. Sequencing was performed on a DNBSEQ-G400 sequencer (MGI, Shenzhen, China). For each sample, we obtained 700–1200 Mb of data.

### 2.4. Bioinformatics

**Figure 2:**
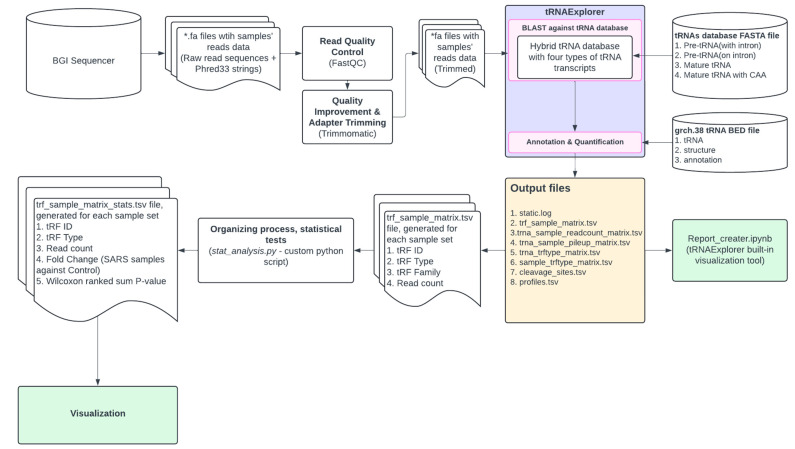
NGS data analysis pipeline. *.fa—FastQ file.

A general design of the bioinformatic processing pipeline is presented in Figure 2.

#### 2.4.1. Read Quality Control

For quality control, we applied FastQC [8]. All datasets qualified quality control standards. Each FastQ file contained between 17 and 27 million reads.

#### 2.4.2. Adapter Trimming

Adapter trimming was executed using Trimmomatic [9]. The long clipping sequence was set as AGTCGGAGGCCAAGCGGTCTTAGGAAGACAA. We opted to use optimal run settings for SE (single-end) reads (TruSeq3-SE:2:30:10 LEADING:3 TRAILING:3 SLIDINGWINDOW:4:15 MINLEN:20), which allowed us to minimise execution time with no data loss. As an input, trimmomatic takes the adapter.fa file, in which our adapter sequence is registered. For each FastQ file, 50 to 68% of the input reads were marked as dropped after running Trimmomatic.

#### 2.4.3. Read Mapping, Count and Normalisation

Full genome alignment and transriptome-based pseudoalignment methods are not applicable for small-RNA-seq identification due to tRNA cleavage events, which led us to utilising “BLAST” (Blast Local Alignment Search Tool) [10].

We implemented BLAST through “tRNAExplorer” [11], a Python-based pipeline, optimised for tRF-profile analysis. In order to utilise tRNAExplorer, we needed a *.bed genome annotation file as a reference, which we would BLAST our reads *.fa files against. While there was an option to generate a custom annotation file, we used an already compiled grch.38 database which came within the tRNAExplorer package. To run the pipeline, we created project directories with sample lists individually for each type of cell and custom configuration file, with data directories and paths and launch options. While tRNAExplorer supports trimming and QC, we omitted these steps due to previous data processing. As an input, each run of the pipeline takes all the *.fa files with read data from the specified directory, list of samples, and path to database *.bed file. We automated these runs with another custom Python script. As a final result of data processing, we obtained *.csv and *.tsv text files, containing BLAST run results for every sample, cleavage sites data and a collective read count table for all provided samples with annotations.

These data were already sufficient for differential expression analysis and visualisation, but we applied another extra step, running a custom Python script which organises data and performs statistical tests.

### 2.5. Statistics

Wilcoxon–Mann–Whitney test was used to perform statistical analysis on the data. *p*-values less than 0.05 were considered statistically significant. The *p*-values for the RNAseq volcano graphics were log converted to log10 (1.31) for data visualisation. All RAW tRF reads and sequences are provided in the Supplementary Materials section.

## 3. Results

### 3.1. tRFs’ Length Distribution in Main Cell Types

**Figure 3:**
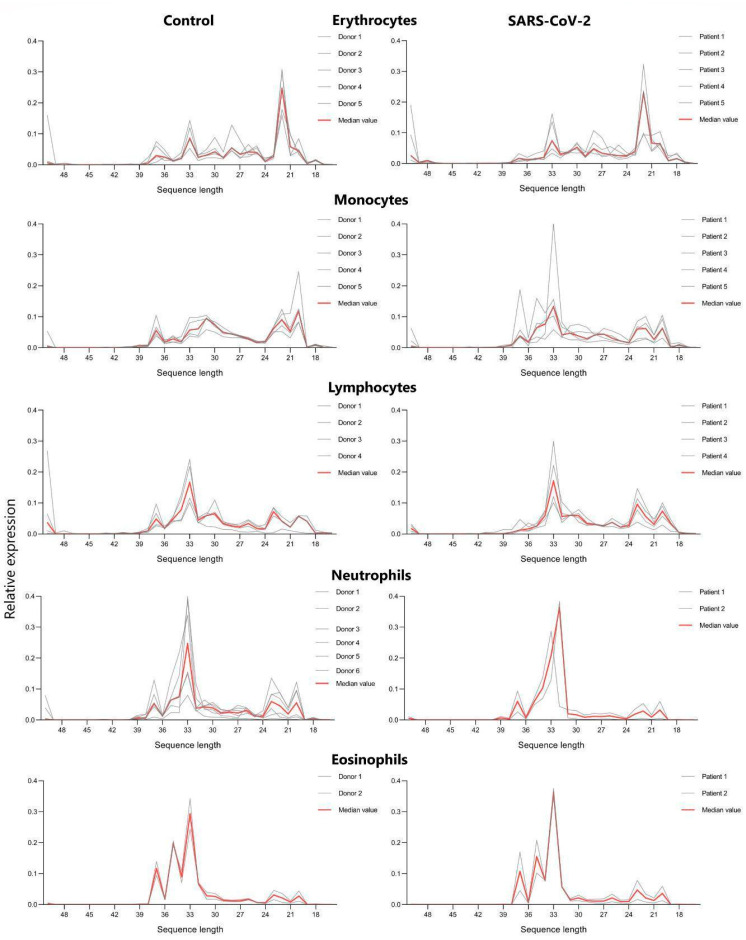

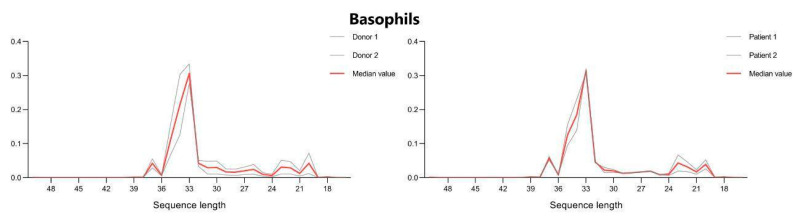
tRF length distribution in main cell types (red line represents median value). Relative expression (y-axis) represents the number of reads with a specific length relative to the number of all tRNA reads in the sample.

Analysing tRFs’ length distribution, we observed a certain similarity between control and severe COVID-19 patients’ erythrocytes, as well as lymphocytes, eosinophils and basophils (Figure 3). In erythrocytes, we observed major spikes between 21 and 23 nucleotides in both control and COVID-19 cells and a minor spike at 33. In lymphocytes, there were major spikes at 32–35 nucleotides and minor spikes at 19–20 and 22–23. For eosinophils, we observed major spikes at 32–35 and minor spikes at 35 and 37. For basophils, we observed major spikes at 32–34 and minor spikes at 20 and 37. Regarding monocytes and neutrophils, we observed an altering pattern: in monocyte control cells, the major spike was at 20, 22 and 31 nucleotides and minor spikes were at 22 and 29-33. In severe COVID-19 patients, monocytes showed major spikes at 33 and minor spikes at 20, 22-23 and 37. In controls, for neutrophils, major spikes appeared to be at 33 and minor spikes at 23, 20 and 37. For neutrophils from COVID-19 patients, there were major spikes at 34 and minor spikes at 20, 22 and 37.

### 3.2. tRF Type Composition in Main Cell Types

**Figure 4:**
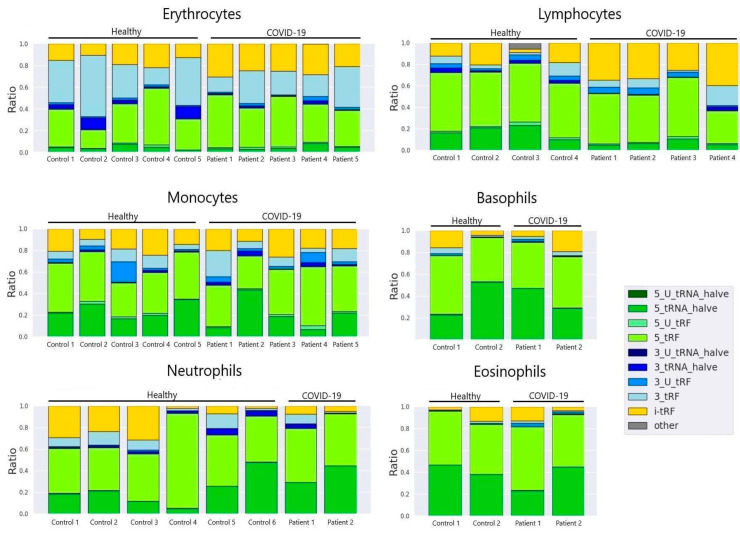
tRF type composition in main cell types (colour matches tRF types).

Analysing the main tRF types in sorted lymphocytes of control donors and severe COVID-19 patients, we observed the prevalence of tRF-5 in 7 out of 8 samples. One severe COVID-19 patient exhibited the presence of i-tRF and tRF-3 types (Figure 4) dominating above all. It is worth noting that this patient had extremely high levels of IL-6-2398 (pg/mL) on the same day that this sample was taken. In all cell types, the most prevalent tRF type appears to be 5-tRF. In erythrocytes, we observed the abundance of 3-tRFs, whereas in other cells, they are represented in a lesser quantity. Additionally, in erythrocytes, we observed that the occurrence of 5 tRNA-halves seem to be lower than in other cell types.

### 3.3. tRF Expression in Control Donors and Severe COVID-19 Patients

**Figure 5:**
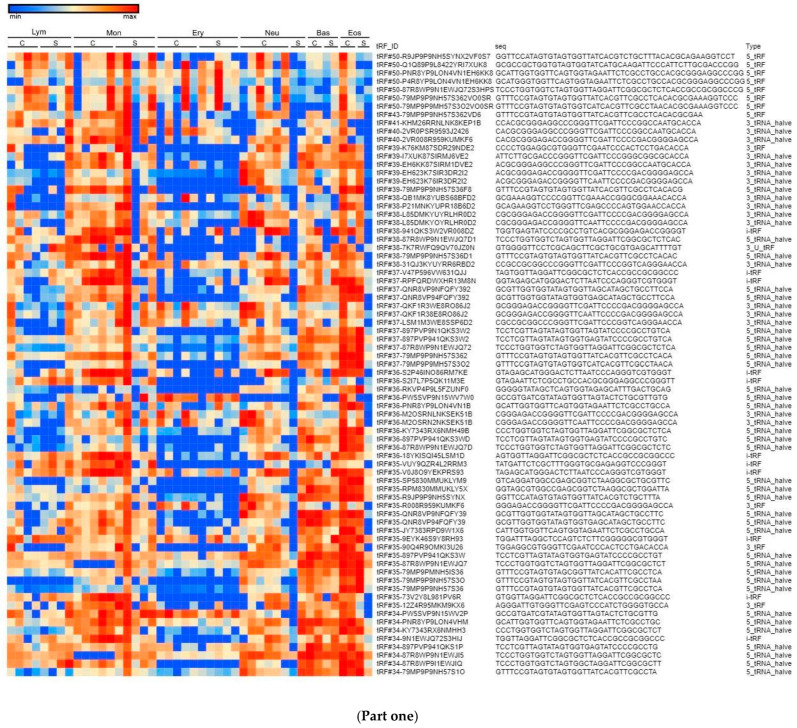

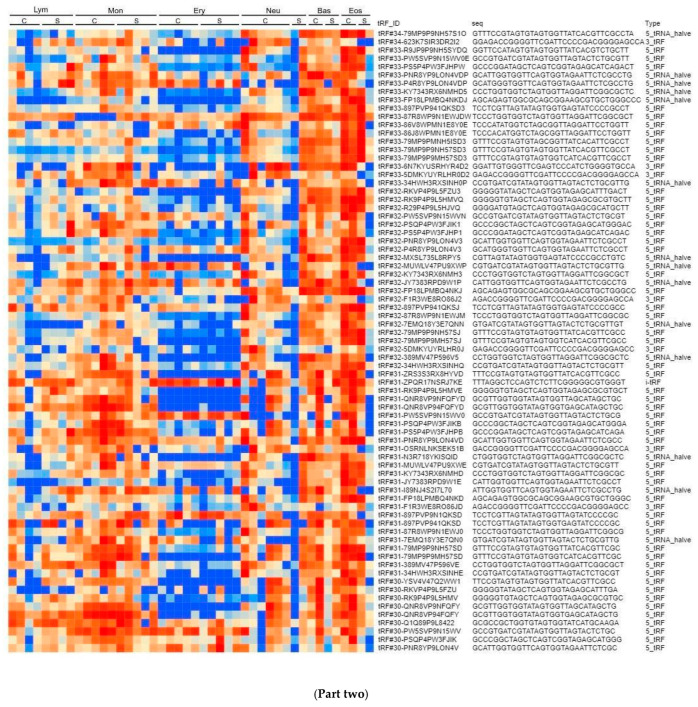
(**Part one**) tRF expression in control donors (C) and severe COVID-19 (S) patients. (Lym—lymphocytes, Mon—monocytes, Ery—erythrocytes, Neu—neutrophils, Bas—basophils, Eos—eosinophils). (**Part two**) tRF expression in control donors (C) and severe COVID-19 (S) patients. (Lym—lymphocytes, Mon—monocytes, Ery—erythrocytes, Neu—neutrophils, Bas—basophils, Eos—eosinophils).

To compare tRF expression in different cell types, we calculated the 150 most abundant tRFs based on RAW read counts, log normalised for data presentation. The heatmap demonstrates a relatively vivid frontier for erythrocytes (Figure 5, Part one, Part two).

### 3.4. tRFs’ Differential Expression in Erythrocytes

**Figure 6:**
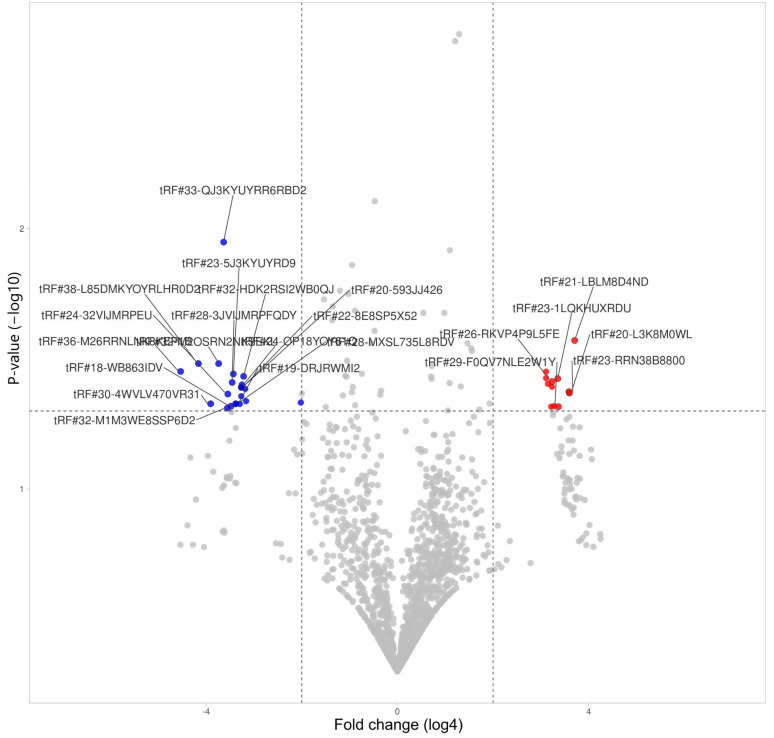
tRF differential expression in erythrocytes (For all volcano plots, we applied log4 (instead of log2) for fold change presentation).

Analysing the expression of tRFs in sorted erythrocytes, we observed 12 upregulated and 21 downregulated fragments (Figure 6, Table 1). tRF#33-QJ3KYUYRR6RBD2 stood out with significant downregulation; however, there are no data on the involvement of this fragment in any known biological processes.

### 3.5. tRFs’ Differential Expression in Lymphocytes

**Figure 7:**
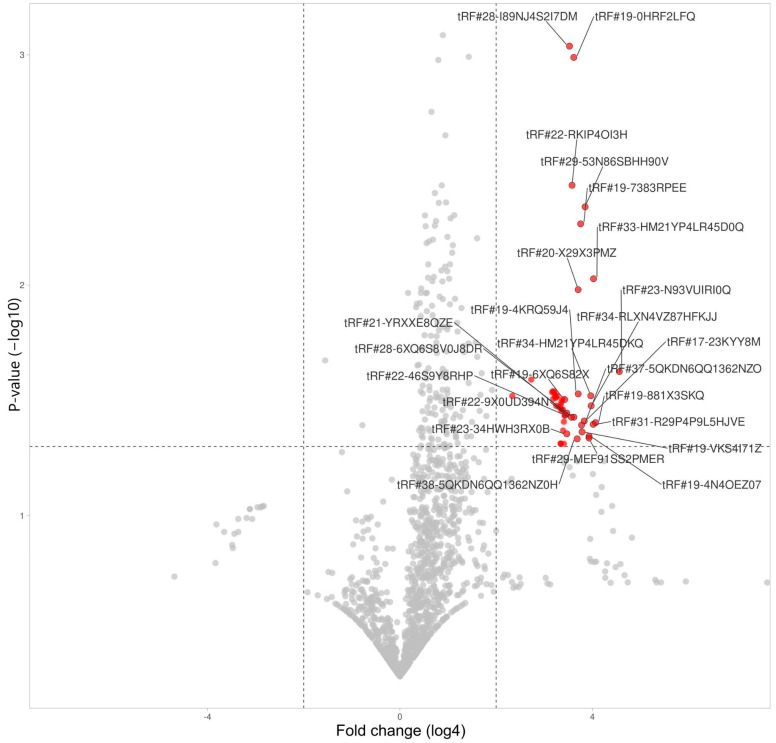
tRFs’ differential expression in lymphocytes.

tRFs in lymphocytes demonstrated significant upregulation with 49 upregulated fragments, and zero downregulated, which vividly sets lymphocytes apart from other cells (Table 2). We applied log4 transformation for data presentation (Figure 7).

### 3.6. tRFs’ Differential Expression in Monocytes

**Figure 8:**
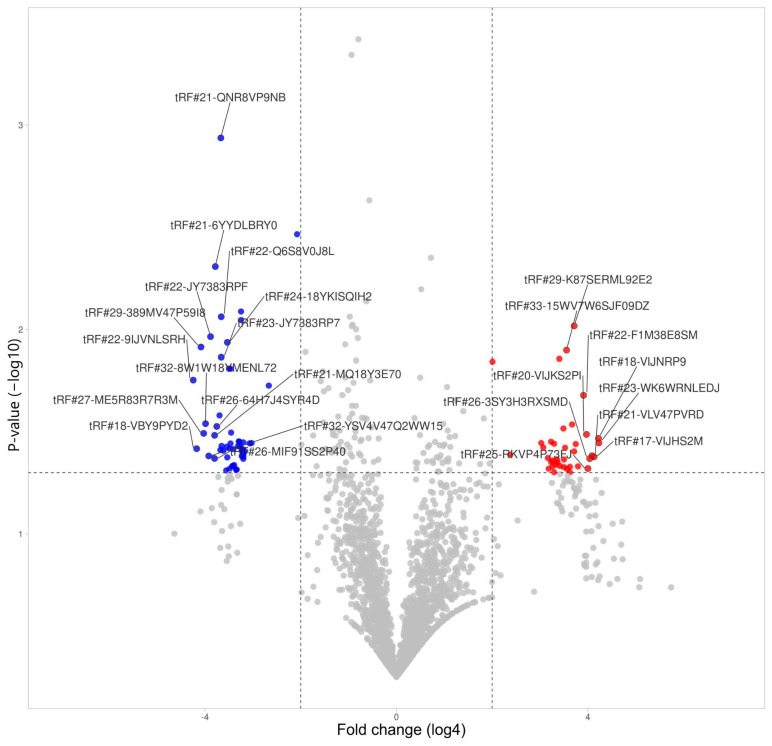
tRFs’ differential expression in monocytes.

Monocytes were presented with the largest overall amount of significant hits: 38 upregulated tRFs and 53 downregulated (Figure 8, Table 3).

## 4. Discussion

The aim of this pilot study was to demonstrate a distinguishable tRF pattern for different sorted cell types. Given the fact that tRFs have been discovered relatively recently, research aimed at understanding their origin can help elucidate their biological purposes.

Analysing tRFs’ length distribution, we observed a certain similarity between control and severe COVID-19 patients’ erythrocytes, lymphocytes, eosinophils and basophils (Figure 3). In the results, we demonstrate the characteristics of length distribution spikes in monocytes and neutrophils, which seem to be altered in comparison to the aforementioned cell types. Monocyte control cells demonstrate high spikes at 31, 22 and 20 nucleotides, whilst the monocytes of SARS-CoV-2-infected patients demonstrate a dominant major spike at 33 nucleotides. In neutrophils, we observed a major spike at 33 nucleotides in control cells and a major spike at 32 in the cells of SARS-CoV-2-infected patients. It is important to state how lymphocytes, one of the key players in the cytokine storm, demonstrate no significant differences in tRF length or tRF types (Figure 3 and Figure 4).

Regarding tRF type composition, it has been previously noted that 3-tRFs are produced in response to various cellular stresses like oxidative stress, hypoxia, and viral infection [12]. Some 3-tRFs can inhibit viral replication by interfering with viral gene expression or packaging. They can act as signalling molecules to mediate stress responses [6,13].

Given the fact that IL-6 is a key mediator of the “cytokine storm” that leads to acute respiratory distress syndrome (ARDS) and multi-organ dysfunction in severe COVID-19 [14], it can be assumed that such a distribution of tRF types may be due to the condition the patient underwent. Other cell types did not exhibit any significant alterations in tRF composition.

Overlooking tRF expression data on our heatmap (Figure 5), we noticed a distinguishable pattern of tRF expression which differs depending on cell type. Erythrocytes are presented as the most distinguishable group of cells in terms of tRF length and tRF expression patterns, which may be due to the fact that the erythrocyte is an inactive cell in terms of synthetic processes. It is worth noting how the expression is increased in the majority of tRFs in granulocytes (neutrophils, basophils, eosinophils). A similar tendency towards increased expression is observed in monocytes. Granulocytes and monocytes both originate from CFU-GM (Colony Forming Unit–Granulocyte–Macrophage), also known as the granulocyte–macrophage progenitor (GMP) [15]. The aforementioned finding suggests that tRFs may dominantly persist from the progenitor stages of cell development and are only slightly modified by environmental or physiological factors.

Most knowledge today regarding tRFs is concentrated around cancer research [2,16,17,18].

In our study, we encountered fragments which have been previously associated with several types of cancer.

***Lymphocytic tRFs:*** It is noted that tRF#31-R29P4P9L5HJVE previously has been acknowledged as a marker for lung cancer prediction among smokers 10 years prior to being diagnosed [19].

tRF#19-VKS4I71Z has been mentioned as an abundant novel trf in a 2016 study of RNA-seq data from human prostate tissue [20].

In a 2023 study dedicated to studying tRFs in cancer, a high-level tRF#34-5QKDN6QQ1362HQ has been mentioned as a predictor of improved survival of breast cancer [2].

***Monocytic tRFs:*** tRF#22-WEK6S1852 in previous research was found to be significantly downregulated and associated with human malignant mesothelioma [16].

A 2019 colon cancer study revealed significantly differentially expressed tRFs between colon cancer tissues and peritumour tissues, whereas another upregulated tRF that we observed—tRF#22-9LON4VN11–was mentioned, demonstrating downregulation in colon cancer tissues with a log2 fold change of −1.26 [17].

We observed another fragment, tRF#18-HSQSD2D2, the downregulation of which has been previously associated with early-stage breast cancer [2].

In a study devoted to examining the dysregulation of different tRFs in chronic lymphocytic leukaemia, tRF-20-RK9P4P9L was amongst the top 15 differentially expressed sRNAs in aggressive chronic lymphocytic leukaemia vs. normal controls, with the linear fold change being −76.64 and −258.08, respectively. Samples were composed of CD5+/CD19+ B cells. It is worth noting that in our study, the same tRF was also significantly downregulated but only in CD14++ CD16− monocytes [18].

tRF#22-WEPSJR852 was found in peripheral blood of fibromyalgia patients in a dissertation dedicated to the search of morphological substrate in fibromyalgia [21].

***Erythrocytes*** appeared to be the only cell type which showed differential expression of tRFs that have not previously been associated with any type of cancer or disease. The fact that the erythrocyte is an inactive cell in terms of synthetic processes may elucidate these findings; however, regarding different cell types, tRFs and their interrelations with the conditions mentioned earlier, it is more likely to treat this as a coincidence, rather than some significant finding.

Differences in the length, type and composition of specific tRFs between different cells are supposedly due to the specificity of the tRNA cleavage system. The same can be said about changes that occur during the cytokine storm. The regulation system of tRNA processing is still poorly understood.

Nonetheless, we can say that it occurs at several “levels”, the first being the regulation of nucleotide modifications in certain molecules [22]. Another stage is dependent on the regulation of RNAses that cleave tRNAs at specific sites.

The main types of nucleases involved in tRF biogenesis are as follows: Dicer—cleaves 5′ ends of mature tRNAs. Angiogenin (ANG)—cleaves mature tRNAs at anticodon loops. RNase Z/ELAC2—cleaves 3′ trailer sequences of pre-tRNAs [11,23,24].

RNase P: excision of external transcribed spacer (ETS) and internal transcribed spacers (ITS) from pre-tRNA transcripts [25].

Regulation of these enzymes can occur at the level of transcription initiation, as well as post-translational modifications of the enzyme

It is also likely that tRNA cleavage is regulated by proteins that bind this molecule [26] and make certain sites inaccessible for the aforementioned process. Unfortunately, we cannot state by what mechanism the difference between tRFs in blood cells is regulated.

In summary, it should be noted that tRF profiles significantly differ in different types of blood cells and demonstrate dramatic differential expression (sometimes more than 500-fold) during the cytokine storm. Such profound differences suggest a major role of tRNA-derived fragments in the functioning of blood cells.

## 5. Conclusions

The composition of tRFs in erythrocytes, monocytes, lymphocytes, neutrophils, basophils and eosinophils was analysed on the basis of sRNA-SEQ. We demonstrate notable alterations in the length and types of these molecules in main blood cell populations. We additionally observed a significant change in the profile of tRFs in the erythrocytes, monocytes and lymphocytes of patients infected with SARS-CoV-2.

## Figures and Tables

**Table 1:** List of differentially expressed tRNA-derived fragments in erythrocytes during severe COVID-19.

tRF Id	Change	Fold Change	*p*-Value
tRF#21-LBLM8D4ND	Increased	170	0.027
tRF#23-RRN38B8800	Increased	146	0.043
tRF#20-L3K8M0WL	Increased	144	0.042
tRF#29-F0QV7NLE2W1Y	Increased	106	0.048
tRF#23-1LQKHUXRDU	Increased	104	0.038
tRF#21-SRRN2EK8B	Increased	94	0.048
tRF#26-RKVP4P9L5FE	Increased	88	0.039
tRF#17-M3WE8SN	Increased	88	0.040
tRF#21-8PR9DM3WE	Increased	86	0.048
tRF#23-6KK87SIRD4	Increased	78	0.039
tRF#33-73NK7F6Z2DNLDW	Increased	74	0.035
tRF#31-79MP9P9MH57SD	Increased	74	0.037
tRF#36-M26RRNLNK8KEP1B	Decreased	536	0.035
tRF#38-L85DMKYOYRLHR0D2	Decreased	320	0.033
tRF#30-4WVLV470VR31	Decreased	224	0.047
tRF#32-M2OSRN2NKSEKL	Decreased	178	0.033
tRF#33-QJ3KYUYRR6RBD2	Decreased	154	0.011
tRF#32-M1M3WE8SSP6D2	Decreased	138	0.049
tRF#24-32VIJMRPEU	Decreased	136	0.043
tRF#18-WB863IDV	Decreased	124	0.048
tRF#28-3JVIJMRPFQDY	Decreased	120	0.039
tRF#23-5J3KYUYRD9	Decreased	116	0.036
tRF#19-DRJRWMI2	Decreased	108	0.047
tRF#28-MXSL735L8RDV	Decreased	108	0.047
tRF#29-YP9LON4VN1EM	Decreased	96	0.047
tRF#20-593JJ426	Decreased	92	0.041
tRF#22-8E8SP5X52	Decreased	92	0.041
tRF#20-PW5SVP9N	Decreased	92	0.044
tRF#24-OP18YOY61Q	Decreased	90	0.040
tRF#32-HDK2RSI2WB0QJ	Decreased	86	0.037
tRF#21-1MQ8YUY60	Decreased	82	0.041
tRF#37-WYL1M3WE8S68L52	Decreased	80	0.046
tRF#28-3K76IR3DR2DV	Decreased	16	0.047

**Table 2:** List of differentially expressed tRNA-derived fragments in lymphocytes during severe COVID-19.

tRF Id	Change	Fold Change	*p*-Value
tRF#23-N93VUIRI0Q	Increased	557	0.024
tRF#19-881X3SKQ	Increased	280	0.039
tRF#33-HM21YP4LR45D0Q	Increased	265	0.009
tRF#31-R29P4P9L5HJVE	Increased	263	0.040
tRF#34-RLXN4VZ87HFKJJ	Increased	247	0.033
tRF#34-HM21YP4LR45DKQ	Increased	245	0.030
tRF#29-MEF91SS2PMER	Increased	233	0.046
tRF#19-4N4OEZ07	Increased	233	0.045
tRF#29-53N86SBHH90V	Increased	207	0.005
tRF#17-23KYY8M	Increased	203	0.039
tRF#19-VKS4I71Z	Increased	190	0.043
tRF#37-5QKDN6QQ1362NZO	Increased	188	0.040
tRF#19-7383RPEE	Increased	183	0.005
tRF#19-4KRQ59J4	Increased	170	0.030
tRF#20-X29X3PMZ	Increased	170	0.010
tRF#38-5QKDN6QQ1362NZ0H	Increased	165	0.046
tRF#28-6XQ6S8V0J8DR	Increased	150	0.037
tRF#19-0HRF2LFQ	Increased	150	0.001
tRF#22-RKIP4OI3H	Increased	143	0.004
tRF#22-9X0UD394N	Increased	140	0.037
tRF#28-I89NJ4S2I7DM	Increased	133	0.001
tRF#21-YRXXE8QZE	Increased	123	0.036
tRF#23-34HWH3RX0B	Increased	123	0.044
tRF#22-46S9Y8RHP	Increased	118	0.037
tRF#19-P7M84I2Q	Increased	115	0.037
tRF#19-6XQ6S82X	Increased	115	0.031
tRF#20-1QKS3W2V	Increased	113	0.049
tRF#19-MIF91S2H	Increased	113	0.039
tRF#21-WRD81H93E	Increased	110	0.043
tRF#21-9L5H52NL0	Increased	110	0.035
tRF#22-W60XY9BIQ	Increased	108	0.031
tRF#23-R9J89O9N9	Increased	105	0.035
tRF#19-8NWE6WIZ	Increased	105	0.035
tRF#28-Z3R918VBY9DV	Increased	105	0.032
tRF#23-9M8O90Q4DZ	Increased	103	0.049
tRF#22-Z3FJ6KEWH	Increased	103	0.049
tRF#23-9N1QKS3WD1	Increased	103	0.033
tRF#22-7EMQ18Y31	Increased	98	0.034
tRF#21-7O3B1NR8E	Increased	93	0.030
tRF#22-282K63ZNQ	Increased	90	0.033
tRF#18-7383RP7	Increased	90	0.031
tRF#23-RPM8309M0F	Increased	88	0.030
tRF#22-6LQ6S8V02	Increased	88	0.030
tRF#35-S4I7LZM3Q01M3K	Increased	85	0.029
tRF#25-QSD2NSWWDZ	Increased	83	0.031
tRF#23-1E6SF8WOD9	Increased	83	0.029
tRF#22-2EJ1OWZIQ	Increased	80	0.029
tRF#34-5QKDN6QQ1362HQ	Increased	44	0.026
tRF#35-5QKDN6QQ1362NZ	Increased	25	0.030

**Table 3:** List of differentially expressed tRNA-derived fragments in monocytes during severe COVID-19.

tRF Id	Change	Fold Change	*p*-Value
tRF#23-WK6WRNLEDJ	Increased	352	0.036
tRF#18-VIJNRP9	Increased	348	0.034
tRF#17-VIJHS2M	Increased	306	0.042
tRF#21-VLV47PVRD	Increased	288	0.041
tRF#26-3SY3H3RXSMD	Increased	270	0.043
tRF#25-RKVP4P73FJ	Increased	256	0.048
tRF#20-VIJKS2PI	Increased	246	0.033
tRF#22-F1M38E8SM	Increased	226	0.021
tRF#44-K8HJ83ML5F82NZD7HY	Increased	192	0.047
tRF#25-RKILQ673FJ	Increased	180	0.036
tRF#28-R1RXQ678Y2D8	Increased	172	0.039
tRF#29-K87SERML92E2	Increased	172	0.010
tRF#31-ZPQR16ZSIJ7KE	Increased	162	0.029
tRF#22-WEK6S1852	Increased	152	0.047
tRF#23-K8HJ83MLDS	Increased	152	0.050
tRF#18-VIJKS2DU	Increased	140	0.048
tRF#33-15WV7W6SJF09DZ	Increased	138	0.013
tRF#25-P21MNKYUPR	Increased	132	0.038
tRF#38-Y2R79K3BEE4O3Q03	Increased	128	0.043
tRF#24-PS5U8918JP	Increased	128	0.047
tRF#21-W60XY9BIE	Increased	126	0.030
tRF#30-6Q46D6PUMZQZ	Increased	114	0.046
tRF#22-9LON4VN11	Increased	112	0.014
tRF#34-IEWS7YRR50SRIZ	Increased	106	0.045
tRF#18-HSQSD2D2	Increased	104	0.043
tRF#32-ZPQR17NSRJ7KQ	Increased	102	0.046
tRF#26-R3HJ83RPFQE	Increased	96	0.036
tRF#19-K876IR19	Increased	96	0.050
tRF#37-9EUK46S9Y8RH93Q	Increased	92	0.044
tRF#20-9VWVEH93	Increased	92	0.047
tRF#28-WS3V2VR0PSDZ	Increased	88	0.035
tRF#20-SVKKN27F	Increased	88	0.044
tRF#32-FN5KYUSRYWRSJ	Increased	82	0.048
tRF#27-WN1Q18Y3HRK	Increased	80	0.042
tRF#20-3K7SIR3D	Increased	70	0.038
tRF#22-WEPSJR852	Increased	66	0.036
tRF#21-MXSL73VLE	Increased	27	0.041
tRF#25-QNR8ZP9LON	Increased	16	0.014
tRF#22-9IJVNLSRH	Decreased	360	0.018
tRF#18-VBY9PYD2	Decreased	326	0.038
tRF#29-389MV47P59I8	Decreased	288	0.012
tRF#27-ME5R83R7R3M	Decreased	266	0.032
tRF#32-8W1W18YMENL72	Decreased	252	0.029
tRF#32-YSV4V47Q2WW15	Decreased	230	0.042
tRF#22-JY7383RPF	Decreased	218	0.011
tRF#26-MIF91SS2P40	Decreased	194	0.043
tRF#21-MQ18Y3E70	Decreased	194	0.033
tRF#21-6YYDLBRY0	Decreased	190	0.005
tRF#26-64H7J4SYR4D	Decreased	182	0.030
tRF#21-MUWLV47PE	Decreased	168	0.026
tRF#21-R84QPYVMD	Decreased	164	0.039
tRF#21-QNR8VP9NB	Decreased	162	0.001
tRF#22-Q6S8V0J8L	Decreased	160	0.009
tRF#23-JY7383RP7	Decreased	160	0.014
tRF#33-1MN0YU09FKRFD2	Decreased	158	0.037
tRF#22-9P9NH57SJ	Decreased	150	0.039
tRF#23-RXSINHZ4DV	Decreased	138	0.049
tRF#24-941QKS3WF8	Decreased	136	0.037
tRF#20-J87383RP	Decreased	134	0.042
tRF#24-18YKISQIH2	Decreased	134	0.012
tRF#38-ML5F924ZDRJKW4DZ	Decreased	128	0.016
tRF#21-9P4P9L5HE	Decreased	126	0.048
tRF#24-Z3RQ18YJFH	Decreased	124	0.038
tRF#23-J87383RP7	Decreased	122	0.036
tRF#20-RK9P4P9L	Decreased	122	0.016
tRF#22-MQ18Y3E7M	Decreased	120	0.032
tRF#28-RUPLQVNRDF0E	Decreased	116	0.047
tRF#22-73H3RXPLM	Decreased	114	0.046
tRF#24-94SX73V2KK	Decreased	112	0.038
tRF#21-MIF91SS20	Decreased	110	0.046
tRF#24-04SXQ3V2KJ	Decreased	104	0.048
tRF#21-9LV470JPD	Decreased	102	0.048
tRF#20-1PSJPM17	Decreased	98	0.037
tRF#34-JY7383RPD9W1JV	Decreased	96	0.035
tRF#24-YDLBRY73JL	Decreased	94	0.035
tRF#21-3P47M26YB	Decreased	92	0.037
tRF#22-6YR29P4PP	Decreased	90	0.008
tRF#21-WB8689SVD	Decreased	90	0.009
tRF#21-WLV47PU9E	Decreased	88	0.039
tRF#21-N1EH6KK80	Decreased	88	0.042
tRF#18-SR99RHD2	Decreased	86	0.038
tRF#33-5F924ZDRJKW4DZ	Decreased	86	0.039
tRF#22-5721V98B3	Decreased	84	0.036
tRF#26-MY73H3RXPL0	Decreased	84	0.040
tRF#22-J4S2I7L7M	Decreased	84	0.042
tRF#28-RKVP4P9L5F0Q	Decreased	84	0.043
tRF#26-94SL735FVI0	Decreased	80	0.039
tRF#18-6M0Y1MY	Decreased	70	0.036
tRF#24-MY73H3RXII	Decreased	66	0.036
tRF#33-1N3KYUSR681SD2	Decreased	40	0.019
tRF#25-R8VP9NFQFY	Decreased	18	0.003

## Data Availability

Data is contained within the article or supplementary material.

## Supplementary Materials

The following supporting information can be downloaded at: https://www.mdpi.com/article/10.3390/life14101294/s1, All RAW read data and nucleotide composition of tRFs are presented in two supplementary files: “Control cells” for control donors and “COVID cells” for patients with severe COVID-19.

## References

[1] Kim V.N. (2005). Small RNAs: Classification, Biogenesis, and Function. Mol. Cells.

[2] Fu M., Gu J., Wang M., Zhang J., Chen Y., Jiang P., Zhu T., Zhang X. (2023). Emerging Roles of tRNA-Derived Fragments in Cancer. Mol. Cancer.

[3] Krishna S., Raghavan S., DasGupta R., Palakodeti D. (2021). tRNA-Derived Fragments (tRFs): Establishing Their Turf in Post-Transcriptional Gene Regulation. Cell. Mol. Life Sci..

[4] Wu W., Choi E.-J., Wang B., Zhang K., Adam A., Huang G., Tunkle L., Huang P., Goru R., Imirowicz I. (2022). Changes of Small Non-Coding RNAs by Severe Acute Respiratory Syndrome Coronavirus 2 Infection. Front. Mol. Biosci..

[5] Yu X., Xie Y., Zhang S., Song X., Xiao B., Yan Z. (2021). tRNA-Derived Fragments: Mechanisms Underlying Their Regulation of Gene Expression and Potential Applications as Therapeutic Targets in Cancers and Virus Infections. Theranostics.

[6] Gong M., Deng Y., Xiang Y., Ye D. (2023). The Role and Mechanism of Action of tRNA-Derived Fragments in the Diagnosis and Treatment of Malignant Tumors. Cell Commun. Signal..

[7] Kondratov K.A., Artamonov A.A., Nikitin Y.V., Velmiskina A.A., Mikhailovskii V.Y., Mosenko S.V., Polkovnikova I.A., Asinovskaya A.Y., Apalko S.V., Sushentseva N.N. (2024). Revealing Differential Expression Patterns of piRNA in FACS Blood Cells of SARS-CoV−2 Infected Patients. BMC Med. Genom..

[8] Andrews S. (2010). FastQC: A Quality Control Tool for High Throughput Sequence Data. http://www.bioinformatics.babraham.ac.uk/projects/fastqc/.

[9] Bolger A.M., Lohse M., Usadel B. (2014). Trimmomatic: A Flexible Trimmer for Illumina Sequence Data. Bioinformatics.

[10] Altschul S.F., Gish W., Miller W., Myers E.W., Lipman D.J. (1990). Basic Local Alignment Search Tool. J. Mol. Biol..

[11] Sun Z., Tan J., Zhao M., Peng Q., Zhou M., Zuo S., Wu F., Li X., Dong Y., Xie M. (2021). Integrated Genomic Analysis Reveals Regulatory Pathways and Dynamic Landscapes of the tRNA Transcriptome. Sci. Rep..

[12] Zhang S., Yu X., Xie Y., Ye G., Guo J. (2023). tRNA Derived Fragments: A Novel Player in Gene Regulation and Applications in Cancer. Front. Oncol..

[13] Kumar P., Kuscu C., Dutta A. (2016). Biogenesis and Function of Transfer RNA Related Fragments (tRFs). Trends Biochem. Sci..

[14] McGonagle D., Sharif K., O’Regan A., Bridgewood C. (2020). The Role of Cytokines Including Interleukin-6 in COVID-19 Induced Pneumonia and Macrophage Activation Syndrome-Like Disease. Autoimmun. Rev..

[15] Guzzi N., Cieśla M., Ngoc P.C.T., Lang S., Arora S., Dimitriou M., Pimková K., Sommarin M.N.E., Munita R., Lubas M. (2018). Pseudouridylation of tRNA-Derived Fragments Steers Translational Control in Stem Cells. Cell.

[16] Filetti V., La Ferlita A., Di Maria A., Cardile V., Graziano A.C.E., Rapisarda V., Ledda C., Pulvirenti A., Loreto C. (2022). Dysregulation of microRNAs and tRNA-Derived ncRNAs in Mesothelial and Mesothelioma Cell Lines after Asbestiform Fiber Exposure. Sci. Rep..

[17] Xiong W., Wang X., Cai X., Xiong W., Liu Y., Li C., Liu Q., Qin J., Li Y. (2019). Identification of tRNAderived Fragments in Colon Cancer by Comprehensive Small RNA Sequencing. Oncol. Rep..

[18] Veneziano D., Tomasello L., Balatti V., Palamarchuk A., Rassenti L.Z., Kipps T.J., Pekarsky Y., Croce C.M. (2019). Dysregulation of Different Classes of tRNA Fragments in Chronic Lymphocytic Leukemia. Proc. Natl. Acad. Sci. USA.

[19] Umu S.U., Langseth H., Zuber V., Helland Å., Lyle R., Rounge T.B. (2022). Serum RNAs Can Predict Lung Cancer up to 10 Years Prior to Diagnosis. eLife.

[20] Olvedy M., Scaravilli M., Hoogstrate Y., Visakorpi T., Jenster G., Martens-Uzunova E.S. (2016). A Comprehensive Repertoire of tRNA-Derived Fragments in Prostate Cancer. Oncotarget.

[21] Erbacher C. (2023). Systemic and Local Mechanisms of Small Fiber Pathology in Female Patients with Fibromyalgia Syndrome. Ph.D. Thesis.

[22] Lyons S.M., Fay M.M., Ivanov P. (2018). The Role of RNA Modifications in the Regulation of tRNA Cleavage. FEBS Lett..

[23] Kazimierczyk M., Wojnicka M., Biała E., Żydowicz-Machtel P., Imiołczyk B., Ostrowski T., Kurzyńska-Kokorniak A., Wrzesinski J. (2022). Characteristics of Transfer RNA-Derived Fragments Expressed during Human Renal Cell Development: The Role of Dicer in tRF Biogenesis. Int. J. Mol. Sci..

[24] Liu B., Cao J., Wang X., Guo C., Liu Y., Wang T. (2021). Deciphering the tRNA-Derived Small RNAs: Origin, Development, and Future. Cell Death Dis..

[25] Li Z., Stanton B.A. (2021). Transfer RNA-Derived Fragments, the Underappreciated Regulatory Small RNAs in Microbial Pathogenesis. Front. Microbiol..

[26] Karanasios E., Simos G. (2010). Building Arks for tRNA: Structure and Function of the Arc1p Family of Non-Catalytic tRNA-Binding Proteins. FEBS Lett..

