# Supplementary material for: Changes in the Repertoire of tRNA-Derived Fragments in Different Blood Cell Populations
Source: Life (Basel). 2024 Oct 12;14(10):1294. doi: 10.3390/life14101294 (PMC11509557; doi:10.3390/life14101294)
Supplement: Supplementary file 1 [file life-14-01294-s001.zip › supl COVID clinical data.pdf]

	**Patient 1**	**Patient 2**	**Patient 3**	**Patient 4**	**Patient 5**
IL-6 (pg/ml)	61,8	204,6	62,54	399	2 398
CRP (C-reactive protein) mg/L	106,7	45,1	105,7	58,9	10,2
Red blood cells (RBC)10^12 /L	3,18	4,29	4,69	3,72	3,77
Lymphocytes (LYMPH)10 ^9/L	2,73	0,72	1,03	0,71	0,65
Monocytes (MONO)10^ 9/L	1,73	0,19	0,5	0,45	0,11
Neutrophils (NEUT)10^9 /L	9,61	7,77	11,16	1,04	3,16
Eosinophils (EOS)10^9/ L	0,33	0,19	0	0	0,03
Basophils (BASO)10^9 /L	0,03	0,01	0,01	0,01	0,01
